# Bile Salt Inhibition of Host Cell Damage by *Clostridium Difficile* Toxins

**DOI:** 10.1371/journal.pone.0079631

**Published:** 2013-11-11

**Authors:** Charles Darkoh, Eric L. Brown, Heidi B. Kaplan, Herbert L. DuPont

**Affiliations:** 1 The University of Texas School of Public Health, Division of Epidemiology, Human Genetics and Environmental Sciences, Center For Infectious Diseases, Houston, Texas, United States of America; 2 The University of Texas Graduate School of Biomedical Sciences, Houston, Texas, United States of America; 3 The University of Texas Medical School, Houston, Texas, United States of America; 4 St. Luke's Episcopal Hospital, Houston, Texas, United States of America; 5 Baylor College of Medicine, Houston, Texas, United States of America; Columbia University, United States of America

## Abstract

Virulent *Clostridium difficile* strains produce toxin A and/or toxin B that are the etiological agents of diarrhea and pseudomembranous colitis. Treatment of *C. difficile* infections (CDI) has been hampered by resistance to multiple antibiotics, sporulation, emergence of strains with increased virulence, recurrence of the infection, and the lack of drugs that preserve or restore the colonic bacterial flora. As a result, there is new interest in non-antibiotic CDI treatments. The human conjugated bile salt taurocholate was previously shown in our laboratory to inhibit *C. difficile* toxin A and B activities in an *in vitro* assay. Here we demonstrate for the first time in an *ex vivo* assay that taurocholate can protect Caco-2 colonic epithelial cells from the damaging effects of the *C. difficile* toxins. Using caspase-3 and lactate dehydrogenase assays, we have demonstrated that taurocholate reduced the extent of toxin B-induced apoptosis and cell membrane damage. Confluent Caco-2 cells cultured with toxin B induced elevated caspase-3 activity. Remarkably, addition of 5 mM taurocholate reduced caspase-3 activity in cells treated with 2, 4, 6, and 12 µg/ml of toxin B by 99%, 78%, 64%, and 60%, respectively. Furthermore, spent culture medium from Caco-2 cells incubated with both toxin B and taurocholate exhibited significantly decreased lactate dehydrogenase activity compared to spent culture medium from cells incubated with toxin B only. Our results suggest that the mechanism of taurocholate-mediated inhibition functions at the level of toxin activity since taurocholate did not affect *C. difficile* growth and toxin production. These findings open up a new avenue for the development of non-antibiotic therapeutics for CDI treatment.

## Introduction

*Clostridium difficile* infection (CDI) is the most common definable cause of hospital-acquired and antibiotic-associated diarrhea in the United States [1]. Pathogenic strains of *C. difficile* possess a 19.6 kb pathogenicity locus responsible for the production of toxin A (308 kDa) and toxin B (269 kDa). These toxins are crucial to *C. difficile* pathogenesis [2], [3], [4], [5], [6], such that strains that do not produce either of these toxins are not associated with disease. Both toxins have similar enzymatic cleavage activities [7], [8], [9] and are cytotoxic to cultured cells; however, toxin B is 100-1,000-fold more potent than toxin A [5], [10], [11]. During infection, these toxins are released into the intestinal lumen where they bind to surface receptors on colonic epithelial cells via their receptor-binding domain and are then internalized by host cells via receptor-mediated endocytosis [12], [13]. The acidic environment within the endosomes activates the cysteine protease activity of the toxins, which cleaves and releases the glucosyltransferase domain located at the N-terminus into the cytosol of the mammalian host [14], [15], [16], [17], [18]. The glucosyltransferase domain monoglucosylates low molecular weight GTPases of the Rho family (RhoA, B, C, Rac, and Cdc42) in the cytosol using cellular uridine diphosphoglucose (UDP-glucose) as the glucose donor [8], [11]. This monoglucosylation interrupts the normal function of the Rho GTPases leading to various deleterious effects including apoptosis, inflammation, cell rounding, actin cytoskeleton dysregulation, and altered cellular signaling [8], [11], [14], [19], [20].

Primary bile salts (cholate and chenodeoxycholate) are biosynthesized from cholesterol in the liver and are conjugated with either glycine or taurine prior to their release into the gall bladder [21], [22], [23]. Conjugation makes bile salts less hydrophobic, more soluble, and prevents passive re-absorption as they traverse the gastrointestinal tract [24]. In addition to their role in fat digestion and absorption, bile salts also inhibit bacterial overgrowth in the small intestine [25], [26], a major site of absorption of nutrients and other metabolites. Consequently, various bacteria synthesize hydrolases that modify conjugated bile salts by deconjugation. Further alterations can occur through dehydroxylation, dehydrogenation, and sulfation, resulting in the generation of secondary and tertiary bile salts [22], [27], [28], [29]. These bacterial modifications render bile salts essentially insoluble, resulting in decreased aqueous concentrations and bacteriostaticity. Moreover, the release of amino acids as a result of these modifications may also act as alternative electron acceptors in this anaerobic environment [30], [31], [32], improving bacterial growth. Bile-salt hydrolases are produced by several genera of enteric bacteria including *Clostridium*, *Bifidobacterium, Bacteroides*, *Lactobacillus*, and *Enterococcus* [29]. We suggest that modifications to bile salts have evolved to enable the intestinal microbiota to gain a survival advantage by counteracting this host defense mechanism.

The foundation of CDI is the ability of this multiple antibiotic-resistant bacterium to overpopulate the human gastrointestinal tract following reduction of the normal gut microbiota by antibiotic therapy [33], [34]. It is well documented that antibiotic treatment is the greatest risk factor for CDI [35], [36], [37], [38], [39]. A large number of *C. difficile* isolates have shown an alarming pattern of resistance to the majority of antibiotics currently used in hospitals and outpatient settings [40], [41], [42], [43]. The dwindling number of antibiotics available to effectively clear CDI and prevent recurrence has sparked new interest in identifying and developing alternative non-antibiotic treatments, either as stand-alone therapies or as adjunctive therapies designed to augment the efficacy of the current antibiotic regimens. The proposed non-antibiotic treatments include: infusion of stool from healthy donors [44], [45], [46], [47], adjunctive use of monoclonal antibodies specific to the toxins [48], probiotics [49], and use of non-toxigenic *C. difficile* strains to out-compete toxigenic strains [50], [51], [52]. As the toxins play an essential role in *C. difficile* pathogenesis, inhibition of either toxin production or toxin activity is another promising approach.

Taurocholate, a major human conjugated bile salt, was previously reported by our laboratory to inhibit the *in vitro* substrate cleavage activity of the *C. difficile* toxins A and B in a dose-dependent manner [53]. In the present study, we demonstrate that physiologic concentrations of taurocholate protect Caco-2 colonic epithelial cells from the damaging effects of the toxins. These findings open up a new avenue that could be harnessed for the development of non-antibiotic therapeutic treatments for CDI.

## Materials and Methods

### Bacterial Strains and Growth Conditions

Active toxin producing *C. difficile* strains VPI 10463 [ATCC (American Type Culture Collection) 43255 (*tcdA*+ *tcdB*+)], 630 [ATCC BAA-1382 (*tcdA*+ *tcdB*+)], 5325 [ATCC BAA-1875 (*tcdA*+ *tcdB*+)], VPI 11186 [ATCC 700057 (*tcdA*− *tcdB*+)], and the hypervirulent strain ATCC BAA-1805 (*tcdA*+ *tcd*B+) were purchased from the ATCC (Manassas, VA). Brain heart infusion (BHI) medium was purchased from Becton Dickinson (Cockeysville, MD). The bacteria were grown in either liquid culture in BHI medium or on Cdifftox Agar plates [54] and incubated anaerobically in an atmosphere of 85% N_2_, 10% H_2_, and 5% CO_2_ at 37°C in a Controlled Atmosphere Anaerobic Chamber (PLAS LABS, Lansing, MI). Sodium taurocholate was purchased from Sigma-Aldrich (St. Louis, MO).

### Growth of *C. difficile* Strains in BHI Medium Containing Taurocholate

Overnight cultures (OD_600nm_ = 1.4) of each strain of *C. difficile* were diluted 1∶100 in 30 ml of fresh BHI broth medium in the presence or absence of 5 mM taurocholate and incubated anaerobically at 37°C. Portions of the cultures were removed every 2 hrs to monitor cell growth by optical density measurements at 600 nm. After a 48-hr incubation period, 1.5 ml of respective cultures were centrifuged at 10,000 x g for 10 mins and the supernatants were tested for the presence of toxins A and B using the enzyme-linked immunosorbent (ELISA)-based Wampole *C. difficile* TOX A/B II assay (TechLab, Blacksburg, VA). The culture supernatants were also tested for toxin activity using the Cdifftox activity assay [53]. Briefly, 250 µl of the culture supernatants were incubated with 5 mM of p-nitrophenyl-β-D-glucopyranoside at 37°C for 4 hrs. The cleavage of the substrate by the toxins was measured spectrophometrically at 410 nm.

### Growth of Caco-2 Cells and Treatment with Taurocholate and *C. difficile* Toxins

The Caco-2 cell line (ATCC HTB-37), a human colonic epithelial cell line was purchased from ATCC. The cells were cultured and maintained in Dulbecco's minimal essential medium (DMEM) containing 10% fetal bovine serum in a humidified incubator with 5% CO_2_. No antibiotics were used in the preparation of the media. Cells were grown to confluence in 24-well culture plates (Corning, Corning, NY) in a final volume of 2 ml as described [55], prior to adding taurocholate in the presence or absence of purified *C. difficile* toxin A or B. The cells were visualized by light microscopy every 24 hrs over a 5-day period using an EVIS XL microscope (Advanced Microscopy Group, Bothell, WA). Cells in different treatment groups were evaluated for morphological changes including rounding, cytoskeleton disruption, and cell death resulting from exposure to the *C. difficile* toxins.

The Caco-2 cells were initially treated with different concentrations of taurocholate (1–25 mM) to determine the appropriate amount that could be tolerated. We determined that 5 mM taurocholate was the ideal amount, as it resulted in undetectable morphological differences when compared to the untreated control cells. Furthermore, purified toxins A and B were both tested initially for cytopathic and cytotoxic effects, but the damaging effect of toxin B was more pronounced and remarkable than toxin A. In fact, toxin B is reported to be 100-1,000-fold more potent than toxin A [5], [10], [11]. As a result, we focused the rest of the experiments on toxin B and 5 mM taurocholate.

### Immunoblot Analysis

*C. difficile* toxins A and B were purified from the hyper-toxin producing strain VPI 10463 (ATCC 43255) according to our recently reported method [53]. Purified *C. difficile* toxins A and B (80 µg each) were separated on 6% polyacrylamide electrophoresis (PAGE) gels and transferred onto Immun-Blot PVDF membrane (BioRad, Hercules, CA) using a Trans-Blot cell (BioRad) transfer apparatus. The membrane was blocked overnight in 10 mM Tris buffered saline with 0.05% tween-20 (TBST) containing 5% skim milk. Following blocking, the membrane was incubated with mouse monoclonal antibodies specific for *C. difficile* toxins A or B (Abcam, Cambridge, MA). The Pierce ECL Western Blotting Kit (Thermo Scientific, Rockford, IL) was then used to probe the membrane for the presence of each toxin using an HRP-conjugated goat anti-mouse IgG secondary antibody, followed by incubation with the ECL substrate according to the manufacturer's instructions. The treated membrane was exposed to X-ray film (Molecular Technologies, St Louis, MO) and processed using a Konica film processor (Konica Corporation, Tokyo, Japan).

### Caspase-3 Activity Assay

Confluent Caco-2 cells were incubated for 24 hrs in the presence of toxin B and 5 mM taurocholate. Following the incubation period, the cells were removed from the 24-well plates using the tip of a 1-ml pipette to scrape the attached cells from the bottom of the wells. The cells were then transferred to 1.5 ml microcentrifuge tubes and centrifuged at 6,000 x g to separate the cells from the medium. Detection of caspase-3 activity was performed as described by the manufacturer of the Caspase-3 Colorimetric Kit (Invitrogen, Carlsbad, CA). Briefly, the harvested cells were lysed on ice for 10 min using 50 µl of cell lysis buffer and centrifuged at 10,000 x g for 15 mins. For this assay, 75 µg of protein was incubated with the substrate reagent for 8 hrs at 37°C and the absorbance at 410 nm was measured using the Spectramax Plus spectrophotomer (Molecular Devices, Sunnyvale, CA). A molar extinction coefficient for *p*-nitrophenol of ε = 17700 M^−1^cm^−1^ was used for the calculations of caspace-3 activity [56]. Total lysate protein concentrations were determined using the Pierce BCA Protein Assay Kit (Thermo Fisher Scientific Inc.).

### Lactate Dehydrogenase Assay

The toxicity and membrane integrity of confluent Caco-2 cells incubated with *C. difficile* toxin B in the presence or absence of 5 mM taurocholate was evaluated. The CytoTox-ONE Homogeneous Membrane Integrity Assay (Promega, Madison, WI) was used to determine the activity of lactate dehydrogenase (LDH) in the spent culture medium. This assay is based on the release of cytosolic LDH from cells with damaged cellular membranes. Confluent Caco-2 cell monolayers were incubated with 4, 8, 12, and 24 µg of purified toxin B in the presence or absence of 5 mM taurocholate in a total medium volume of 2 ml in 24-well plates for 24 hrs. The supernatant was tested for LDH activity according to the protocol provided by the manufacturer. Briefly, 100 µL of the CytoTox-ONE Reagent was added to 100 µL of the culture supernatant in a 96-well microtiter plate and incubated at 22°C for 10 min. Following the incubation period, 50 µL of Stop Solution was added to each well. Fluorescence was measured using Spectromax I3 (Molecular Devices, Sunnyvale, CA) at an excitation wavelength of 560 nm and an emission wavelength of 590 nm.

### Data Analysis

All the data were analyzed and plotted using GraphPad Prism version 6.02 for Windows (GraphPad Software, San Diego, California). One-Way ANOVA and Wilcoxon-Mann-Whitney tests were used to compare differences between the samples. In all cases, statistical significance was defined as having a *P* value of <0.05.

## Results

### Purification of the *C. difficile* Toxins

**Figure 1:**
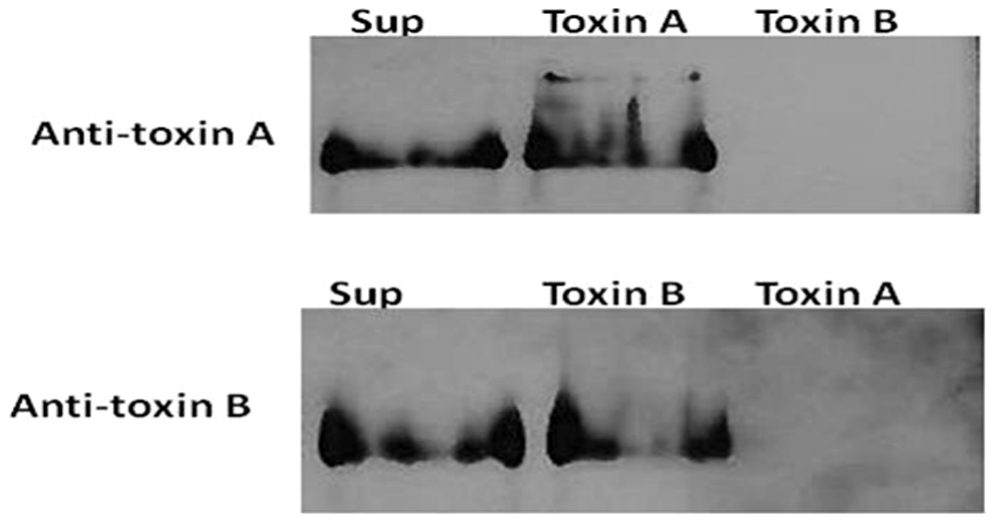
Immunoblot analysis of purified *C. difficile* toxins A and B. Purified proteins (80 µg each) were subjected to 6% PAGE and transferred onto PVDF membranes. Each membrane was probed using monoclonal primary antibodies specific for toxin A or B. The Pierce ECL Western Blotting Kit was used to detect the bound antibodies. The membrane was exposed to X-ray film (Molecular Technologies, St Louis, MO) and processed using a Konica film processor (Konica Corporation, Tokyo, Japan). Sup, crude culture supernatant; Toxin A, purified toxin A; Toxin B, purified toxin B.

To evaluate the effect of *C. difficile* toxins on confluent Caco-2 cells, native A and B toxins were purified from supernatant fluid generated from cultures of the toxin-producing strain VPI 10463 grown in dialysis bags with a 100-kDa molecular weight cut off. The purification steps consisted of DEAE-Sepharose anion exchange and Sephacryl S-300 gel filtration chromatography as previously reported [53]. Analysis of the final purified fractions by PAGE, followed by Coomassie staining confirmed the presence of two single bands in each final fraction (data not shown) corresponding to toxins A and B, respectively, as detected by immunoblot analysis (Fig. 1).

### Taurocholate and Toxin Titration Assays

**Figure 2:**
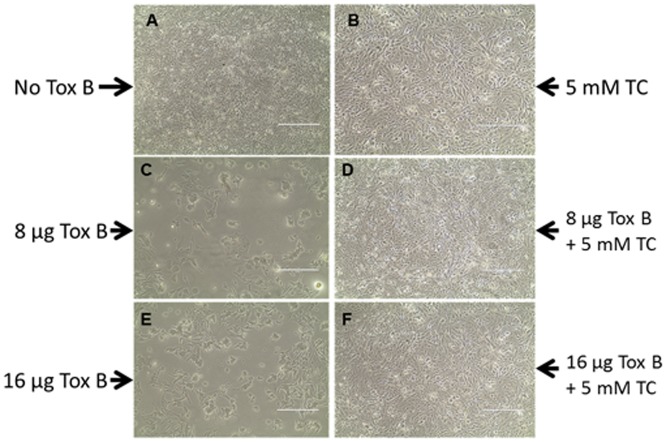
Effect of *C. difficile* toxin B and taurocholate on Caco-2 Cells. Confluent Caco-2 cell monolayers were incubated with 8 and 16 µg of toxin B in the presence or absence of 5 mM taurocholate in a total medium volume of 2 ml in 24-well plates for 24 hrs. Images were captured using an EVIS XL microscope. Magnification 10×. Tox B, purified toxin B; TC, taurocholate.

The total concentration of bile salts in the human small bowel ranges from 2 to 30 mM, depending on diet and other metabolic conditions [57]. To determine the amount of taurocholate that could be tolerated by Caco-2 cells, confluent Caco-2 cells were cultured in the presence of 1 to 25 mM taurocholate, as described in the Materials and Methods. From this analysis, 5 mM of taurocholate was determined to be the optimum concentration tolerated based on cell viability. Confluent cells incubated with and without 5 mM taurocholate were indistinguishable (Fig. 2, A-B). The cells were not viable at taurocholate concentrations above 20 mM (data not shown). Confluent Caco-2 cells were also cultured in the presence of increasing amounts of the toxins (0, 4, 8, 12, 16, and 24 µg) to determine the amount of each toxin needed to elicit visible cytotoxic or cytopathic changes. Both toxins A and B caused damage to the cells. However, cell damage was more pronounced within 24 hrs of incubation with toxin B. The toxin-treated CaCo-2 cells appeared rounded, spindle-like, detached from the plate surface, and presented with an altered cytoskeleton; all of these phenotypes were consistent with previous reports describing the cytopathic and cytotoxic effects of these toxins [19], [20]. Visualization of the cell damage caused by toxin A required longer incubation times of more than 48 hrs compared to that required for toxin B (data not shown). These results confirmed the earlier reports that toxin B is more potent than toxin A [5], [10], [11]. As a consequence, we focused on the more potent toxin B for the Caco-2 protection experiments described.

### Taurocholate Protects Caco-2 Cells from Toxin B-Mediated Toxicity

Taurocholate was shown previously in our laboratory to inhibit the substrate cleavage activity of toxins A and B *in vitro* in a dose-dependent manner [53]. Confluent Caco-2 cells were incubated with toxin B in the presence or absence of 5 mM taurocholate to test whether taurocholate could protect the cells from toxin B-mediated toxicity *ex vivo* (Fig. 2). When a lethal dose of toxin B (16 µg) was added to Caco-2 monolayers in the presence of taurocholate, no detectable cytopathic damage to the cells was apparent (Fig. 2F). A similar protective effect of taurocholate was observed when the cells were also incubated with toxin A (data not shown). These data indicate that taurocholate protects the cells from toxin-mediated damage and supports our previous report [53], which demonstrated that taurocholate inhibits the activities of toxins A and B.

### Taurocholate Decreases *C. difficile* Toxin B-Mediated Induction of Host Cell Caspase-3 Production

**Figure 3:**
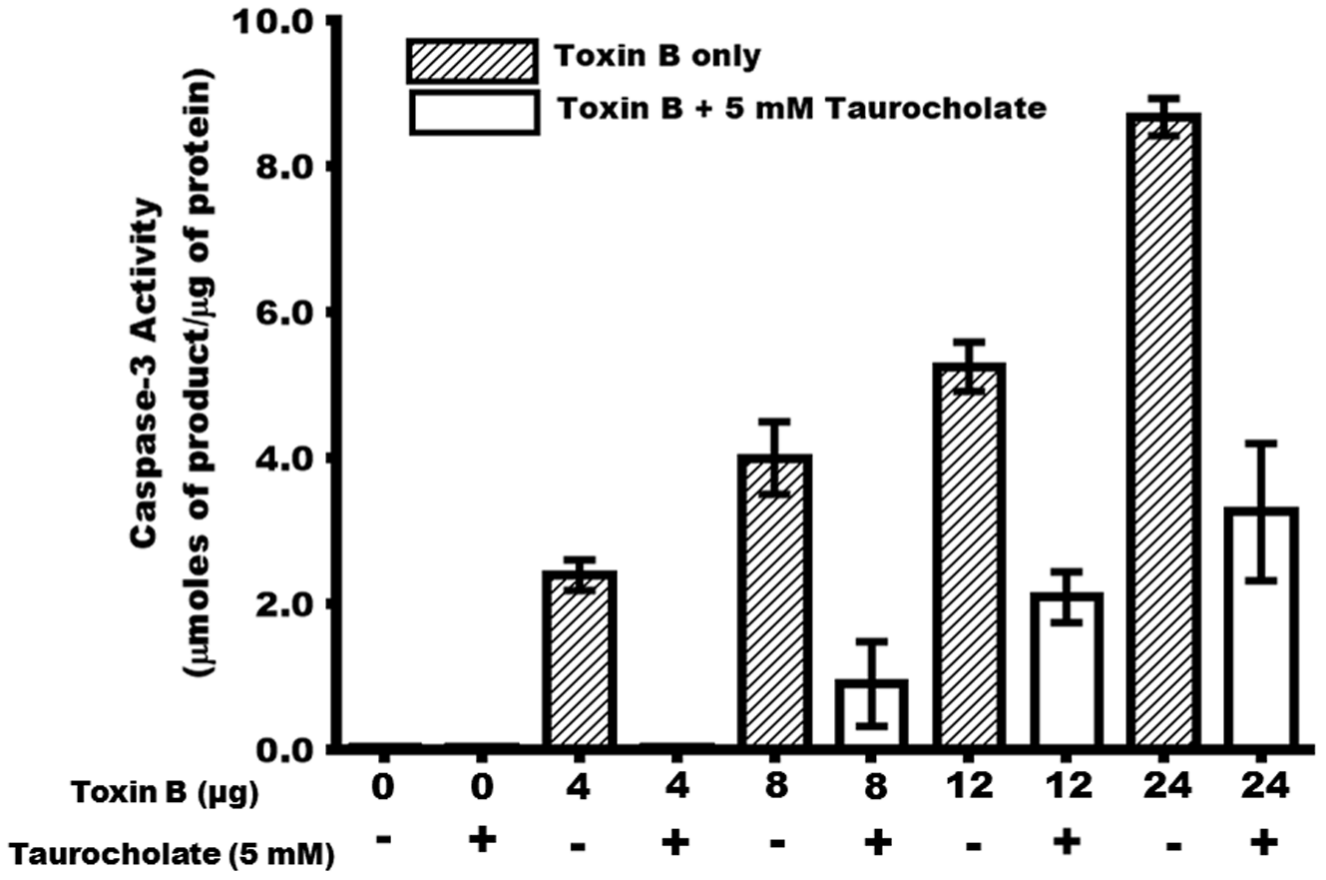
Taurocholate significantly decreases *C. difficile* toxin B-mediated induction of caspase-3 activity in Caco-2 cells. Confluent Caco-2 cells were incubated for 24 hrs with 0, 4, 8, 12 and 24 µg of toxin B in the presence (+) or absence (−) of 5 mM taurocholate. Cell monolayers were scraped from the 24-well plates and lysed to obtain crude protein lysates. Crude protein lysates (75 µg) were incubated with the caspase-3 colorimetric substrate reagent (Invitrogen) for 8 hrs at 37°C and absorbance at 410 nm was measured. A molar extinction coefficient for *p*-nitrophenol of ε = 17700 M^−1^cm^−1^ was used in the calculations of caspace-3 activity [56]. One-Way ANOVA test showed a significant difference (P<0.003) between the caspase-3 activities of the cells cultured with 5 mM taurocholate and the cells cultured without taurocholate. Error bars represents the standard deviation from three replicate experiments.

During *C. difficile* infection, apoptosis is an important downstream effect resulting from receptor-mediated toxin endocytosis into the host cell cytoplasm and subsequent inactivation of the host GTPases. Previous reports showed that toxin A induces cell death in human epithelial cells *ex vivo* via the activation of caspases [58], [59], [60]. Our results indicate that various amounts of toxin B (4, 8, 12, and 24 µg) induced significant caspase-3 activity in Caco-2 cells (Fig. 3). The caspase-3 activation cascade plays a crucial role in several apoptotic mechanisms, including activation of key apoptotic mediators essential to chromatin condensation, DNA fragmentation, dismantling of the cell, and formation of apoptotic bodies [61], [62], [63]. To determine whether taurocholate protected the Caco-2 cells from toxin B damage by preventing apoptosis, caspase-3 activity was assessed in taurocholate-treated and untreated cells. Crude protein lysates prepared from confluent Caco-2 cells incubated with various amounts of toxin B in the presence or absence of taurocholate were tested for caspase-3 activity. In cells cultured without toxin B or taurocholate, no caspase-3 activity was detected. In the presence of toxin B, however, caspase-3 activity was detected in a dose-dependent manner (Fig. 3). Cells cultured with 4–24 µg of toxin B showed a 2.5-7.5-fold increase in caspase-3 activity. In contrast, cells cultured with toxin B in the presence of taurocholate had significant reduction in caspase-3 activity. Remarkably, addition of 5 mM taurocholate reduced caspase-3 activity in cells treated with 4, 8, 12, and 24 µg of toxin B by 99%, 78%, 64%, and 60%, respectively. These data demonstrated that taurocholate protected Caco-2 cells from the damaging effects of toxin B, as evidenced by the decreased levels of the pro-apoptotic protease, caspase-3.

### Taurocholate Impacts the Membrane Integrity of Toxin B-Treated Cells

**Figure 4:**
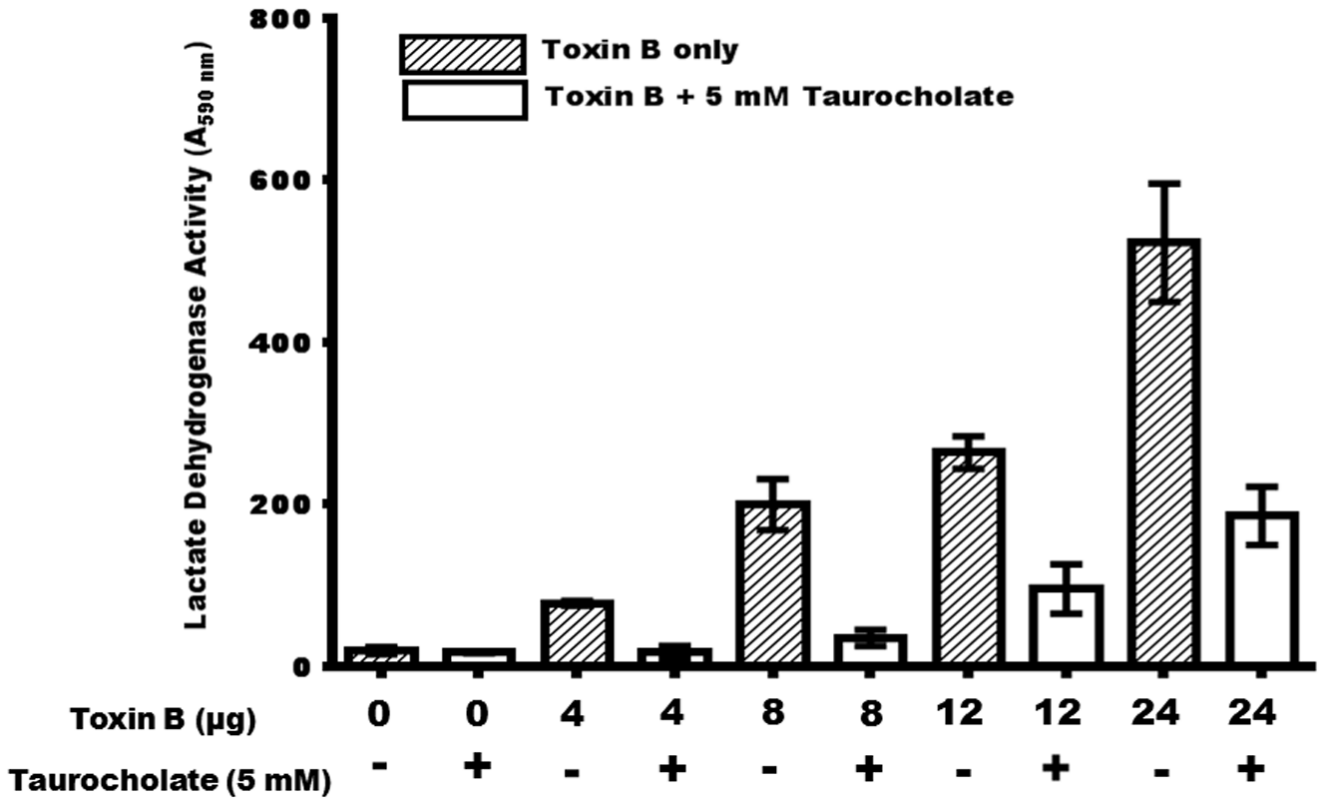
Taurocholate significantly decreases toxin B-mediated Caco-2 cell membrane integrity. Confluent Caco-2 cells were incubated for 24 hrs with 0, 4, 8, 12 and 24 µg of toxin B in the presence (+) or absence (−) of 5 mM taurocholate. The spent culture supernatant fluid (100 µL) was tested for lactate dehydrogenase activity using the CytoTox-ONE Homogeneous Membrane Integrity Assay (Promega). Fluorescence was measured using Spectromax I3 (Molecular Devices) at an excitation wavelength of 560 nm and an emission wavelength of 590 nm. One-Way ANOVA test showed a significant difference (P<0.001) between the lactate dehydrogenase activities of toxin B-treated Caco-2 cells cultured with 5 mM taurocholate and the cells cultured without taurocholate. Error bars represents the standard deviation from three replicate experiments.

The membrane integrity of confluent Caco-2 cells following incubation with toxin B was evaluated in the presence of taurocholate using the CytoTox-ONE Homogeneous Membrane Integrity Assay (Promega). This assay was used to determine the activity of lactate dehydrogenase (LDH) in the medium as a result of toxin B-mediated damage to the cells. LDH is a soluble cytosolic enzyme, which is released into the extracellular medium as a consequence of damage to the cell membrane. The results showed that the membrane integrity of Caco-2 cells incubated with toxin B was compromised in a dose dependent manner, as evidenced by an increased LDH activity (Fig. 4). Interestingly, spent culture medium from confluent Caco-2 cells incubated with both toxin B and taurocholate exhibited significantly less LDH activity compared to spent medium from cells incubated with toxin B only. These results support the morphological defects and cytotoxic effects observed microscopically in the toxin B-treated cells (Fig. 1), and underscore the significance of taurocholate in protecting the cells from toxin damage.

### Taurocholate Inhibits *C. difficile* Toxin Activity with no Significant Effect on Growth or Toxin Production

**Figure 5:**
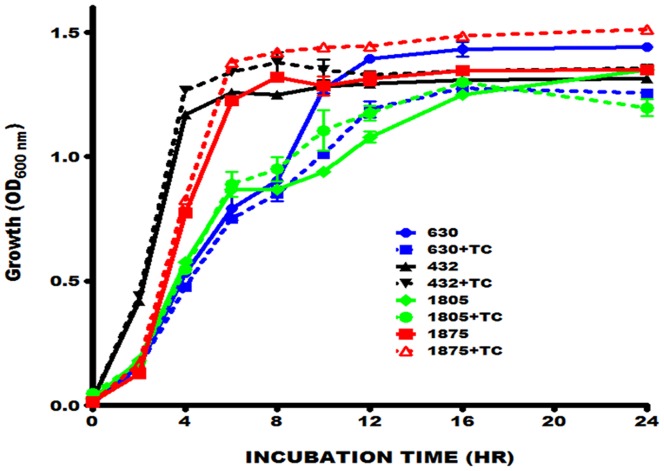
Taurocholate has no significant effect on the growth of *C. difficile* toxin A- and B-producing strains. Overnight cultures (OD _600 nm_ = 1.4) of each strain of *C. difficile* tested were diluted 1∶100 in 30 ml of fresh medium with or without 5 mM of taurocholate (TC) and incubated anaerobically at 37°C for 24 hrs. Portions (2 ml) of the cultures were removed at the indicated times for OD_600 nm_ measurement. Strain designations: 630, ATCC BAA-1382; 432, ATCC 43255; 1805, ATCC BAA-1805; 1875, ATCC BAA-1875. The error bars represent the standard deviation from three different experiments. Mann-Whitney test showed no significant difference between the *C. difficile* cells growth in the presence or absence of 5 mM taurocholate.

In addition to their role in fat digestion and absorption, bile salts also inhibit bacterial overgrowth in the small intestine [25], [26], which is a major site of nutrient and metabolite absorption. Some enteric bacteria such as *E. coli* and *Salmonella* produce various bile salt hydrolases capable of modifying bile salts and rendering them non-toxic to bacteria. Analysis of the *C. difficile* genome revealed the presence of homologues of bile salt hydrolases similar to those characterized in classic enteric bacteria. To examine whether *C. difficile* could grow in the presence of the physiologic taurocholate concentration used, different toxigenic *C. difficile* strains were cultured in the presence of 5 mM taurocholate. Some strains appeared to grow better than others when cultured with taurocholate, however, none of the strains tested exhibited viability defects (Fig. 5).

**Figure 6:**
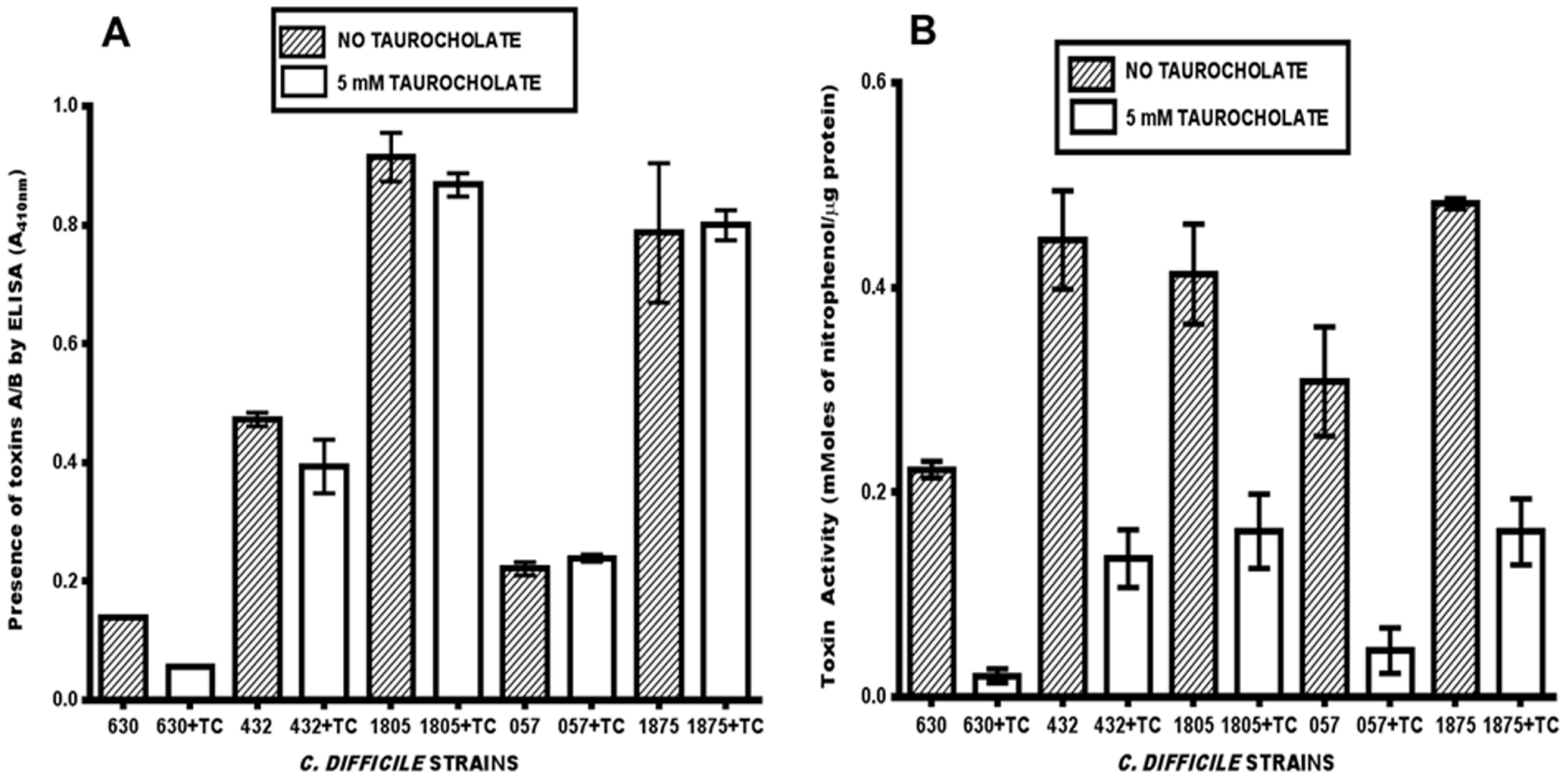
Taurocholate has no effect on *C. difficile* toxin production (A), but inhibits *C. difficile* toxin activity (B). Overnight cultures (OD_600 nm_ = 1.4) of each strain of *C. difficile* tested were diluted 1∶100 in 30 ml of fresh medium with or without 5 mM of taurocholate and incubated anaerobically at 37°C for 48 hrs. The culture supernatant fluids were tested for the presence of toxins by ELISA using the Wampole *C. difficile* TOX A/B II assay and toxin activity using the Cdifftox activity assay [53]. Strain designations: 630, ATCC BAA-1382; 432, ATCC 43255; 1805, ATCC BAA-1805; 057, ATCC 700057; 1875, ATCC BAA-1875. The error bars represent the standard deviation from three different experiments. TC = taurocholate. Mann-Whitney test showed no significant difference [P = 0.333 (630) P = 0.332 (432); P = 0.667 (1805); P = 0.333 (057); P = 0.999 (1875)] in the amount of toxin produced by *C. difficile* cells cultured either with 5 mM taurocholate or without taurocholate. However, toxin activity detected in the culture supernatant fluids of *C. difficile* cells cultured with taurocholate was significantly lower than those cultured without taurocholate.

To assess the effect of taurocholate on toxin production and toxin activity, an ELISA-based assay was used to analyze toxin production and the Cdifftox activity assay [53] was used to determine toxin activity. As shown in Figure 6A, the presence of taurocholate did not affect toxin production. However, toxin activity was significantly lower (P<0.05) in the supernatant fluids of the strains cultured in the presence of taurocholate (Fig. 6B). The addition of 5 mM taurocholate to the *C. difficile* culture medium reduced the total toxin activity differently, depending on the strain: 91% (ATCC BAA-1382), 85% (ATCC 700057), 70% (ATCC 43255), 67% (ATCC BAA-1875), and 61% (ATCC BAA-1805). These results indicate that taurocholate specifically inhibits toxin activity without affecting toxin production, and suggests that bile salts play an important role in the pathogenesis of the *C. difficile* toxins. Furthermore, the presence of genes that encode bile salt hydrolases in the *C. difficile* genomes did not appear to affect the inhibition of *C. difficile* toxin activity by taurocholate.

## Discussion

*Clostridium difficile* is the etiological agent of antibiotic-associated colitis, including pseudomembranous colitis, and the leading definable cause of nosocomial diarrhea. Treatment of *C. difficile* infections (CDI) has been hampered by recurrence of the infection, emergence of strains with increased virulence, sporulation, multi-drug resistance, lack of drugs with superior functional activity in the colon, and the lack of drugs that restore or preserve the colonic microbiota following antibiotic treatment. As a result, there is new interest in finding alternative treatments, either as stand-alone therapies or therapies designed to augment the efficacy of currently used antibiotic regimens. A novel treatment approach would be to inhibit the activities of toxins A and B, which are directly responsible for the intestinal damage and subsequent inflammation associated with CDI. An approach, which targets the toxins without affecting cell growth, may be ideal since it is unlikely to impose selective pressure on *C. difficile*, thereby minimizing the risk of developing resistance. In our search for compounds that inhibit toxin activity we developed the Cdifftox assay [53], which detects the ability of toxins A and B to cleave a chromogenic substrate that is stereochemically similar to their native substrate (UDP-glucose). Using this Cdifftox assay, we identified taurocholate as a compound that inhibits *in vitro* the substrate cleavage activity of these toxins [53].

In this report, we demonstrated that a physiologic concentration of taurocholate (5 mM) protected *ex vivo* confluent human colonic epithelial Caco-2 cells from *C. difficile* toxin B-mediated damage. Taurocholate also protected the cells from toxin A damage (data not shown). When taurocholate and toxin B (16 µg) were added simultaneously to confluent Caco-2 cell monolayers, toxin-mediated cytopathic effects were prevented (Fig. 2). One of the mechanisms by which *C. difficile* toxins mediate cell damage is by inducing apoptosis. Specifically, toxin A has been reported to induce cell death in human epithelial cells *ex vivo* by activating caspases [58], [59], [60]. We demonstrate here for the first time that toxin B induces caspase-3 production in Caco-2 cells in a dose-dependent manner (Fig. 3). Furthermore, toxin B also induced membrane damage in a dose-dependent manner, as evidenced by the elevated lactate dehydrogenase activity in the culture medium (Fig. 4). Remarkably, both caspase-3 and lactate dehydrogenase activities were significantly reduced when toxin B-treated confluent Caco-2 cells were cultured with physiologic concentration of taurocholate. The protective effect of taurocholate was apparent even when the cells were treated with lethal doses of toxin B.

The concentration of taurocholate used in this study did not affect the growth or toxin production of the *C. difficile* strains tested (Fig. 5, 6A). However, it significantly decreased the total toxin activity in the supernatant fluids of all the strains tested (Fig. 6B). We noted that the lowest percent inhibition of toxin activity was observed in the hypervirulent *C. difficile* strains that produce high levels of the toxins, suggesting that more taurocholate may be necessary to neutralize the cells.

The mechanism of taurocholate-mediated inhibition of *C. difficile* toxin activity remains to be determined. Brandes et al.[64] reported that tauroursodeoxycholic acid, a modified conjugated bile acid, induced phosphorylation of Rac1/Cdc42 leading to inhibition of *C. difficile* toxin B-mediated monoglucosylation of this GTPase. Taurocholate may function through hydrophobic interactions to saturate the Caco-2 cell membranes, thereby inhibiting toxin entry and/or toxin activity. Other possible inhibitory mechanisms may involve direct effects of taurocholate on the toxins, such as a structural alteration with subsequent loss of toxin activity, or binding of taurocholate to the toxins leading to the prevention of entry into the host cell. Further research is on-going to identify the mechanism of taurocholate action.

One limitation of this *ex vivo* study was the inability to co-culture *C. difficile* with the Caco-2 cells in the presence of taurocholate; *C. difficile* does not grow under aerobic conditions, and Caco-2 cells do not grow under anaerobic conditions. Thus, an *in vivo C. difficile* animal model is required to provide more insight into how these findings may be exploited to develop a treatment intervention against *C. difficile* infections.

It is important to note that the majority of nutrient absorption in the gastrointestinal tract occurs in the small intestine, where bile salts are at much higher concentrations compared to the colon. This difference in bile salts concentration is due to the reabsorption of more than 95% of the total human bile via the enterohepatic circulation in the ileum [65], which is directly upstream of the colon. Clearly, only a small amount of bile salts enter the colon where *C. difficile* most frequently colonizes. An intriguing explanation for why CDI pathology is mostly limited to the bile salt-deficient colon, and not the bile salt-rich small intestine, is that toxin activity may be inhibited in the small intestine by the high bile salt concentrations. Our data suggest that the *C. difficile* toxins are active in the colon because of its low bile salt concentrations. The therapeutic benefits of bile salts are well documented; they prevent hepatocyte injury and cholestasis [66], [67], [68], drug-induced cholestasis [69], and endotoxin absorption [70], [71]. Our findings highlight the point that bile salt concentration represents a host-mediated mechanism that naturally protects the absorptive surfaces of the small intestine from deleterious microbial products and also acts to inhibit bacterial growth. Thus, the lack of bile salts in the small intestine in diseased states (such as cirrhosis of the liver) may lead to bacterial overgrowth and result in a competition for the essential nutrients required for normal human growth and function.

We suggest that uncovering a mechanism to deliver higher concentrations of bile salts and/or their derivatives, perhaps in conjunction with antibiotics, into the colon of individuals suffering from recurrent CDI may help protect the colon from the damaging effects of the *C. difficile* toxins and facilitate clearance of the pathogen. This line of research may result in a novel treatment of *C. difficile* infections; an option likely to maintain intestinal homeostasis and minimize the risk of drug resistance.
